# Topical Pimecrolimus as a New Optional Treatment in Cutaneous Sarcoidosis of Lichenoid Type

**DOI:** 10.1155/2014/976851

**Published:** 2014-02-03

**Authors:** Antonella Tammaro, Claudia Abruzzese, Alessandra Narcisi, Giorgia Cortesi, Francesca Romana Parisella, Pier Paolo Di Russo, Gabriella De Marco, Severino Persechino

**Affiliations:** ^1^NESMOS Department, UOC Dermatology, Faculty of Medicine and Psychology, University of Rome “Sapienza”, 00189 Rome, Italy; ^2^Faculty of Medicine, University of Towson, Towson, MD 21204, USA

## Abstract

We report the case of cutaneous sarcoidosis of lichenoid type successfully treated with pimecrolimus. For the first time in the literature, we propose the use of this topical calcineurin inhibitor for the treatment of the cases refractory to common therapy regimens.

## 1. Introduction

Lichenoid sarcoidosis is an extremely rare cutaneous manifestation of sarcoidosis, occurring in 1%-2% of all cases and presenting with multiple erythematous or violaceous, slightly scaling maculopapules localized on the trunk, limbs, and face, without systemic involvement. Different therapeutic approaches have been described for the treatment of cutaneous sarcoidosis, but the lesions are often refractory to the common treatments. We described the case of a female patient affected by cutaneous sarcoidosis of lichenoid type successfully treated with topical calcineurin inhibitor Pimecrolimus, never been reported in the literature until now.

## 2. A Case Report

**Figure 1:**
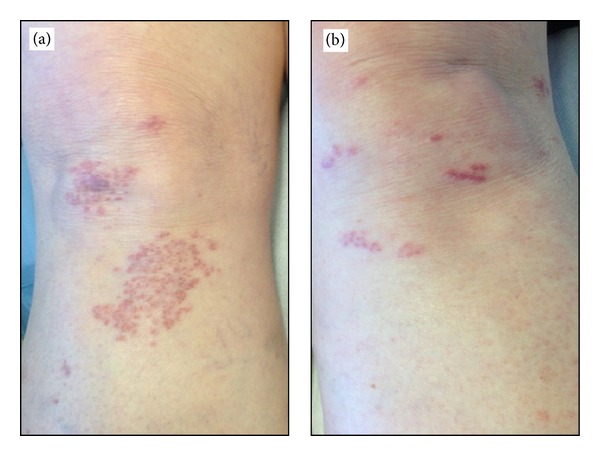
(a) Infiltrated, nonfollicular, lichenoid papules with a tendency to group, located on both knees; (b) a nearly complete remission of the lesions after 6-months therapy with topical 1% pimecrolimus.

A 66-year-old woman was referred to our department with a 6-month history of 1-2 mm diameter, violaceous, nonfollicular, infiltrated lichenoid papules with a tendency to group, located on the right knee (Figure 1(a)). The onset had been abrupt and no antecedent of trauma was found. She had been using a clobetasol ointment once daily for 2 months with no improvement of the lesions.

**Figure 2:**
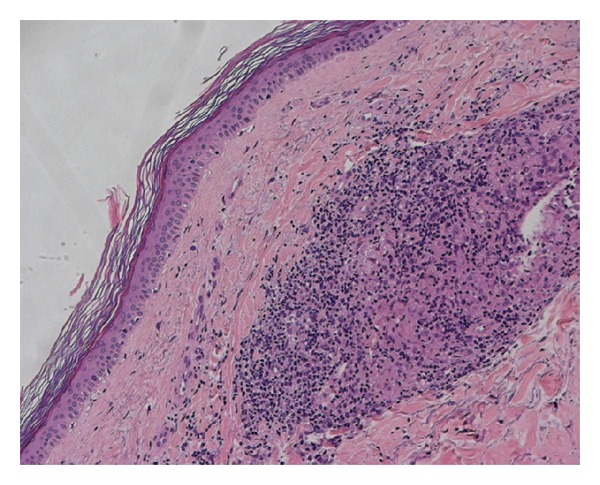
H&E staining, 100x magnification. Epidermis presents rete ridges flattening and orthotopic hyperkeratosis. In the deep dermal layer a sarcoidosis-like granulomatous reaction is visible.

**Figure 3:**
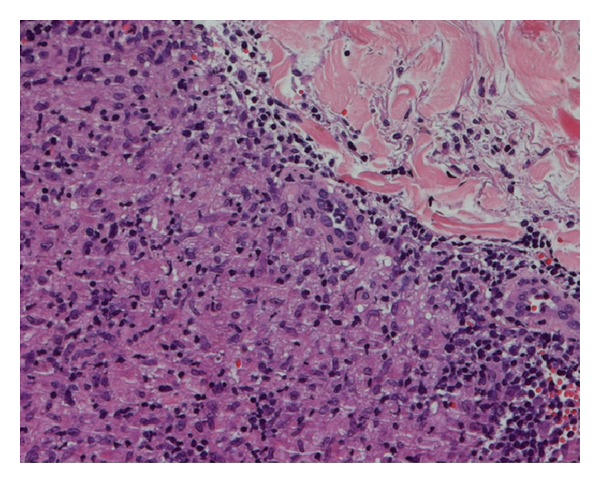
H&E staining, 200x magnification. Sarcoidosis-like granulomas are composed by epithelioid macrophages aggregates, surrounded by a scarce rime of small lymphocytes. Neither asteroid bodies nor Schaumann bodies were observed.

**Figure 4:**
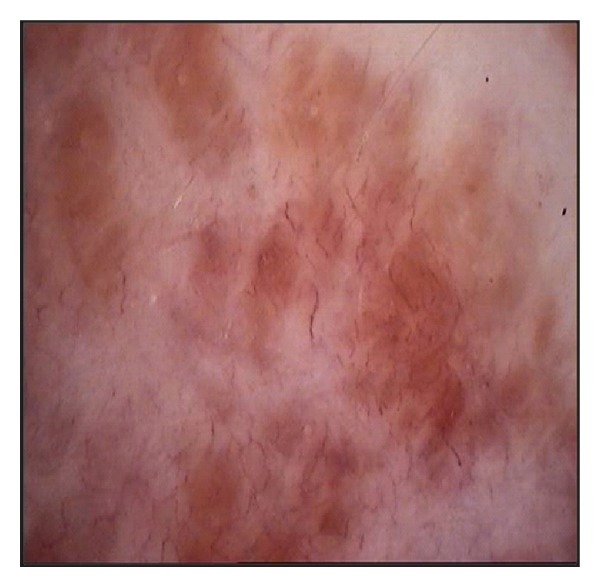
Videodermoscopic features: round to oval yellow-brown lesion with absence of white Wickham striae.

She did not complain of any systemic symptoms or take any kind of drugs. In the past medical history, no relevant diseases were referred, except for a papillary thyroid carcinoma excised 4 years before. General physical examination did not reveal any abnormality or lymphadenopathy. Cutaneous biopsy of one of the lesions revealed mild thinning of the epidermis, and a nonnecrotizing lymphohstiociytic granulomatous infiltrate in the superficial and deep dermis (Figures 2 and 3). Neither eosinophils nor mucin were seen. Gram stain, stain for fungi, and special stains for mycobacteria were negative. We also performed a videodermoscopy evaluation of the lesions, showing a round to oval yellow-brown lesion with absence of white Wickham striae (Figure 4).

Chest X-ray, eye examination, and blood tests, including complete blood count, hepatic and renal functions, serum angiotensin-converting enzyme, blood and urinary calcium, and beta-2-microglobulin, were all normal. A tuberculin reaction was also negative. Clinical and histological findings led to a diagnosis of cutaneous lichenoid sarcoidosis without systemic involvement. As treatment with topical corticosteroids had been ineffective, topical 1% pimecrolimus cream twice daily was prescribed. The patient was followed up monthly and after 6 months of treatment, we noted a nearly complete remission of the lesions on the knee, with no topical and systemic adverse effects referred to by the patient (Figure 1(b)) and no relapses of the lesions at 12-month followup.

## 3. Discussion

Sarcoidosis is a chronic multisystemic granulomatous disease of unknown etiology, characterized by the formation of noncaseating granulomas in the involved organs. Cutaneous involvement is about 25% and the most common clinical manifestations are maculopapular lesions, whereas lupus pernio is the most characteristic skin lesion.

The lichenoid-type lesion is an extremely rare cutaneous manifestation of sarcoidosis and is estimated in 1%-2% of all cases of skin sarcoidosis [1]. Clinically, the lichenoid type presents with multiple 1 to 3 mm, erythematous or violaceous, slightly scaling maculopapules involving an extensive area of the skin. They occur singly or in groups, especially localized on the trunk, limbs, and face. Dermoscopy usually shows round to oval yellow-brown lesion with absence of white Wickham striae; even if this pattern is not specific for sarcoidosis, these homogeneus patches are indicative of a granulomatous skin disease [2].

Lichenoid lesions have been particularly reported in young children, frequently presenting together with eye and joint complications, but respiratory involvement is usually absent [3, 4].

Diverse therapeutic approaches, including topical, intralesional, and systemic corticosteroids, antimalarials, methotrexate, tetracycline, infliximab, thalidomide, allopurinol, and isotretinoin, have been described in the management of cutaneous sarcoidosis; however, their efficacy varies and adverse effects, like the skin atrophy, sometimes limit their use. In the last decade an increasing interest derived from the use of topical calcineurin inhibitors (TCIs) in the treatment of cutaneous sarcoidosis [3, 5, 6].

Tacrolimus is a macrolide immunosuppressive agent isolated from *Streptomyces tsukubaensis* and modulates T-cell-mediated responses by inhibiting calcineurin-dependent dephosphorylation activation of the transcription factor NF-AT [7].

Pimecrolimus is a lipophilic macrolactamic agent isolated from ascomycin; it selectively modulates T-cell-mediated response binding with high affinity to macrophilin-12 and inhibiting calcineurin calcium-dependent phosphatase. These drugs, blocking the production and release of proinflammatory T helper1 cytokines and activation of T lymphocytes, have been successfully used in atopic dermatitis for many years [8].

It is supposed that, in sarcoidosis, granulomas may be caused by a series of factors, including Th1 cell activation by antigen-presenting cells (APC) and their cytokine release (IL2 and interferon-gamma) with a subsequent inflammatory cell recruitment and tissue infiltration; in addition, T lymphocytes and macrophages are responsible for an overproduction of TNF-alpha, amplifying the granulomatous reaction [6].

The ability of tacrolimus to inhibit both hapten-induced production of Th1 cytokines and the production of TNF-alpha by T cells and macrophages constitutes an effective rational for its use in the treatment of sarcoidosis [9]. In the literature are described few cases of cutaneous sarcoidosis treated with tacrolimus and only one case of lichenoid-type sarcoidosis treated with tacrolimus is reported [9–13].

Pimecrolimus has the same actions of tacrolimus but is more selective than tacrolimus in the modulation of immune cells: in fact it acts specifically on T lymphocytes and mast cells with a more antiinflammatory than immunosuppressive action, whereas tacrolimus also downregulates Langerhans cells and basophilic cells [14].

Moreover, pimecrolimus is more lipophilic and binds with higher affinity to structural skin proteins than tacrolimus, having a lower penetration rate in the dermis and a minor systemic biodisponibility [15].

These features allow a better efficacy and tolerability profile than tacrolimus, reducing the possibility of adverse systemic effects [16].

Based on the data in the literature about the use of topical tacrolimus in cutaneous sarcoidosis, we experienced the possibility to use also topical pimecrolimus in the treatment of lichenoid type of cutaneous sarcoidosis, deciding for a long-term treatment of six months for the appearance of relapses with a shorter therapy period, as described in previous cases reported in the literature [3].

The promising results we obtained let us define pimecrolimus as a useful therapeutic option for refractory forms of cutaneous sarcoidosis, besides representing a valuable alternative to tacrolimus for its better efficacy and tolerability profile.
